# Atteinte cutanée et nasale: ne pas méconnaitre la sarcoïdose

**DOI:** 10.11604/pamj.2015.20.183.6304

**Published:** 2015-02-27

**Authors:** Madiha Mahfoudhi, Khaled Khamassi

**Affiliations:** 1 Service de Médecine Interne A, Hôpital Charles Nicolle, Tunis, Tunisie; 2 Service d'ORL. Hôpital Charles Nicolle, Tunis, Tunisie

**Keywords:** sarcoïdose, peau, nez, granulome, sarcoidosis, skin, nose, granuloma

## Image en medicine

La sarcoïdose est une maladie granulomateuse systémique d’éthiopathogénie inconnue. Les localisations ORL sont rares. Le traitement médical de la sarcoïdose nasale doit être administré précocement afin de prévenir l’évolution vers une fibrose locale. Nous rapportons l'observation d'un homme âgé de 50 ans, sans antécédents particuliers, ayant présenté un érythème facial, une obstruction nasale bilatérale et quelques épisodes d’épistaxis évoluant depuis un an. Il n'y avait pas de contage tuberculeux ni de signes respiratoires. L'examen physique a révélé un placard érythémateux infiltré de la joue gauche et de la pyramide nasale donnant un aspect de lupus pernio. L'endoscopie nasale a révélé une muqueuse nasale congestive et croûteuse associée à une hypertrophie turbinale inférieure bilatérale avec un aspect granuleux de la tête des cornets inférieurs. Le cavum et les aires ganglionnaires cervicales étaient libres. A la biologie, il avait une lymphopénie, un syndrome inflammatoire et un dosage de l'enzyme de conversion à 4 fois la normale. Les diagnostics de tuberculose, lymphome et sarcoïdose ont été évoqués. L'intradermo-réaction à la tuberculine était négative. L'examen anatomopathologique de la biopsie cutanée et nasale a retrouvé des lésions granulomateuses épithélio-giganto-cellulaires sans nécrose caséeuse. Le diagnostic d'une sarcoïdose à double localisation nasale et cutanée a été retenu. Le bilan d'extension de la maladie était négatif. Le traitement s'est basé sur une corticothérapie par voie générale (1mg/kg/j) puis dégression progressive associée à une corticothérapie locale (nasale et cutanée). L’évolution était favorable sur le plan clinique et biologique avec un recul de 3 ans.

**Figure 1:**
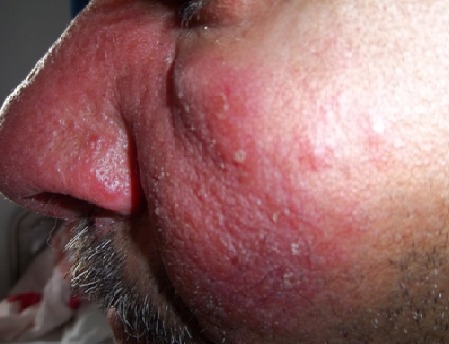
Placard érythémateux cutané de la joue gauche et du nez

